# Taurine Attenuates M1 Macrophage Polarization and IL-1β Production by Suppressing the JAK1/2-STAT1 Pathway via Metabolic Reprogramming

**DOI:** 10.3390/biology14121751

**Published:** 2025-12-06

**Authors:** Zi’an Zhang, Danyue Li, Suhui He, Weilv Xu, Qian Lv, Yumeng Wang, Jinxia Xu, Zexu Yu, Shiyang Liu, Yuanxiang Ge, Fushan Shi, Yuqi Yan

**Affiliations:** Department of Veterinary Medicine, College of Animal Sciences, Zhejiang University, Hangzhou 310058, China; 22317114@zju.edu.cn (Z.Z.); 22217119@zju.edu.cn (D.L.); hsh@njmu.edu.cn (S.H.); wlxu@zju.edu.cn (W.X.); lvqian@zju.edu.cn (Q.L.); 22317053@zju.edu.cn (Y.W.); 12417043@zju.edu.cn (J.X.); zxyu@zju.edu.cn (Z.Y.); 22417108@zju.edu.cn (S.L.); 22417114@zju.edu.cn (Y.G.); sfs@zju.edu.cn (F.S.)

**Keywords:** taurine, macrophage, immunometabolism, spermine, sepsis

## Abstract

Dysregulated macrophage polarization is associated with various diseases, including sepsis, atherosclerosis, and fibrotic diseases. While taurine is known to exert immunomodulatory effects, its mechanism in regulating M1 macrophage polarization and interleukin-1β (IL-1β) production remains incompletely understood. This study aimed to elucidate the role of taurine in modulating macrophage immunometabolism and inflammatory signaling. Using thioglycolate-elicited peritoneal macrophages and macrophage cell lines, we assessed taurine’s impact on lipopolysaccharide (LPS)/interferon-γ (IFN-γ)-induced M1 polarization through metabolomics and a range of molecular biology techniques. Pharmacological manipulation of the JAK1/2-STAT1 pathway and an LPS-induced murine sepsis model were used for mechanistic and therapeutic validation. Our results demonstrate that taurine significantly suppressed M1 polarization. Metabolomic profiling uniquely identified a marked increase in intracellular spermine as a key metabolic alteration induced by taurine. This increased spermine subsequently inhibited JAK1/2-STAT1 activation, leading to reduced IL-1β release. In mice, taurine alleviated systemic inflammation, reduced pathological damage in multiple organs, and decreased intestinal M1 macrophage infiltration. These findings establish a novel mechanism where taurine attenuates M1 polarization and IL-1β production through metabolically driven spermine accumulation and subsequent JAK1/2-STAT1 suppression, highlighting its therapeutic potential for inflammatory diseases.

## Simple Summary

An uncontrolled or dysregulated inflammatory response is a key driver of tissue damage and organ failure in critical illnesses such as sepsis. Taurine, a naturally occurring nutrient, is recognized for its role in supporting immune function. This research reveals a unique mechanism through which taurine helps to mitigate this excessive inflammation. We demonstrate that taurine reprograms the metabolism of immune cells, boosting the production of a protective molecule called spermine. This molecule, in turn, suppresses a key signaling pathway responsible for driving inflammation, leading to reduced levels of a potent inflammatory signal molecule. In a mouse model of sepsis, taurine supplementation effectively reduced inflammation and tissue injury in several organs, including the lungs and intestines. These findings identify taurine as a potent regulator of the immune response and suggest that ensuring adequate taurine intake could be a valuable nutritional strategy for managing inflammatory conditions.

## 1. Introduction

Macrophages, widely distributed throughout the organism, are vital for maintaining homeostasis by orchestrating the two phases of inflammation: a pro-inflammatory phase for threat clearance and a subsequent resolution phase for tissue repair [1]. Upon stimulation, macrophages polarize into distinct functional phenotypes [2]. The pro-inflammatory M1 type induced by LPS/IFN-γ (a full list of abbreviations is provided in Supplementary Table S1), combats infections through mediators like IL-1β and TNF-α [3], and the anti-inflammatory M2 type induced by interleukin-4 (IL-4), which facilitates tissue repair [4]. As exemplified in Table 1, abnormalities in macrophage phenotypic switching are intimately linked to a spectrum of diseases, underscoring the importance of precisely regulating this polarization balance [5,6,7,8]. A full list of abbreviations used is provided in Supplementary Table S1.

Taurine exerts direct immunomodulatory effects, including the upregulation of key anti-inflammatory and tissue-repair mediators such as Chitinase-like protein 3 (Ym1) and Interleukin-10 (IL-10) [9]. Its efficacy is further established in vivo, where it alleviates TNBS-induced colitis in rats by suppressing myeloperoxidase (MPO) activity and oxidative stress [10]. Notably, within inflammatory environments, taurine reacts with hypochlorous acid (HOCl) to form taurine chloramine (TauCl), which exhibits even greater anti-inflammatory potency by inhibiting signaling pathways such as NF-κB [11]. These findings collectively underscore taurine’s importance as a key immunoregulator.

Metabolic reprogramming refers to the remodeling of metabolic patterns in immune cells, regulated by signaling pathways like mTOR and JAK-STAT [12]. Focusing on amino acid metabolism, this process provides biosynthetic precursors and acts as a key signaling node [13]. For instance, M1 macrophages highly express inducible nitric oxide synthase (iNOS) to metabolize arginine for pro-inflammatory functions [14]. Leucine activates mTORC1 through its metabolite acetyl-CoA-mediated acetylation of Raptor [15]. Glutamine metabolism also supports M1 activation by supplying energy and carbon sources [16]. However, the mechanism by which taurine, a derivative of a conditionally essential amino acid, regulates metabolic reprogramming and polarization in macrophages remains unclear.

Thus, we elucidated the role of the JAK1/2-STAT1 pathway in taurine-mediated metabolic reprogramming and IL-1β suppression in M1 macrophages and evaluated taurine’s therapeutic potential in a murine model of LPS-induced sepsis.

## 2. Materials and Methods

### 2.1. Antibodies

Antibodies against IKK (ab32041), NLRP3 (ab214185), JAK2 (ab108596), Phospho-JAK2 (ab32101), and Phospho-STAT1 (ab109461) were obtained from Abcam (Cambridge, UK). Antibodies against JAK1 (IPH0038), Phospho-IKK (IPH0892), AIM2 (IPB0929), and NLRC4 (IPB11308) were obtained from Baijia Sen Biotech (Beijing, China). Antibodies against p65 (10745-1-AP), Phospho-JAK1 (66466-1-Ig), and β-actin (60008-1-Ig) were obtained from Proteintech (Rosemont, IL, USA). The antibody against Phospho-p65 (bs-0982R) was obtained from Bioss (Beijing, China). The antibody against STAT1 was obtained from MedChemExpress (Shanghai, China). The antibody against NALP1 (PA5-17275) was purchased from Thermo Fisher Scientific (Waltham, MA, USA). Secondary antibodies, including goat anti-mouse HRP (FDM007) and goat anti-rabbit HRP (FDR007), were obtained from FUDE Biological Technology (Hangzhou, China). Primary antibodies were diluted according to the manufacturer’s instructions, while the mouse and rabbit secondary antibodies were diluted at 1:25,000 and 1:20,000, respectively.

### 2.2. Isolation of Thioglycolate-Elicited Peritoneal Macrophages (TGPMs)

Two to four days after intraperitoneal injection of 4% thioglycolate, the peritoneal cavity was flushed with phosphate-buffered saline (PBS) to collect macrophages, which were then cultured in complete DMEM medium [17]. Following purification by adhesion, the macrophages were stimulated with LPS (1 μg/mL; Invitrogen, Carlsbad, CA, USA) and IFN-γ (20 ng/mL; Peprotech, Cranbury, NJ, USA) for 12 h to induce M1 polarization [18,19].

### 2.3. Cell Culture and Treatment

RAW 264.7 and THP-1 cells were cultured under standard conditions (37 °C, 5% CO_2_). The growth medium was RPMI 1640 supplemented with fetal bovine serum (FBS) and 1% penicillin/streptomycin. To induce M1 polarization, RAW 264.7 cells and TGPMs were stimulated as indicated. THP-1 cells were first differentiated with PMA (200 ng/mL; Sigma-Aldrich, St. Louis, MO, USA) for 6 h, followed by a 24 h stimulation with LPS (1 μg/mL) and IFN-γ (20 ng/mL). For the taurine-treated groups, taurine (Tau; 10 mmol/L) was added to the medium concurrently with the LPS and IFN-γ stimulants and maintained throughout the 12-h polarization period.

### 2.4. Quantitative PCR Analysis

Total RNA was extracted from cells with the RNA-easy Isolation Reagent (Vazyme Biotechnology, Nanjing, China) and quantified. Using the HiScript III RT SuperMix for qPCR (Vazyme Biotechnology, Nanjing, China), we generated cDNA via reverse transcription. Subsequent quantitative real-time PCR (qRT-PCR) was carried out using the ChamQ Universal SYBR qPCR Master Mix (Vazyme Biotechnology, Nanjing, China). The 2^−ΔΔCT^ method was applied to calculate relative gene expression, using β-actin for normalization.

### 2.5. Enzyme-Linked Immunosorbent Assay

After centrifugation, concentrations of IL-1β and TNF-α in cell culture supernatants and tissue homogenates (from lung, liver, jejunum, and colon) were measured using mouse ELISA kits (Cusabio, Wuhan, China).

### 2.6. Immunoblot

Total proteins were extracted with RIPA lysis buffer (Beyotime, Shanghai, China) supplemented with 1% PMSF (Beyotime, Shanghai, China). After separation by SDS-PAGE and transfer to PVDF membranes, the membranes were blocked and subsequently incubated with primary and secondary antibodies. Protein bands were visualized using an ECL detection system (Clinx Science Instruments, Shanghai, China).

### 2.7. Metabolites Analysis

For the analysis of metabolites in macrophages, TGPMs (1 × 10^7^ cells/well) were first processed as instructed and harvested. Metabolites from the cells were then quenched and extracted. These extracted metabolites were quantified using a gas chromatography-mass spectrometer (GC-MS) by Novogene (Beijing, China).

### 2.8. Animal Experiment

Female ICR mice (3-week-old) were housed in individually ventilated, pathogen-free cages (temperature 20–30 °C, relative humidity 50–60%, lighting cycle 12 h/day) with free access to food and water. They were randomly divided into three groups: blank, LPS, and LPS+Tau. The blank and LPS groups received normal drinking water, whereas the LPS+Tau group was provided with water supplemented with 1% Tau for four weeks. Throughout this period, the body weight of all mice was recorded. Subsequently, mice in the LPS and LPS+Tau groups were intraperitoneally injected with LPS (10 mg/kg) [20], while the blank group received an equivalent volume of PBS. Tissue samples (lung, liver, spleen, kidney, jejunum, colon) were collected and weighed six hours after the LPS challenge. All animal experiments were carried out according to the WMA Statement on animal use in biomedical research. These experiments received approval from the Ethics Committee for Animal Welfare in Experiments, Zhejiang University (ZJU20250777).

### 2.9. Histological Analysis

Following fixation, the lung, spleen, and jejunum tissue samples were processed using a standard histological protocol. Briefly, the tissues were dehydrated through a graded ethanol series, cleared in xylene, and embedded in paraffin wax. The embedded tissues were then sectioned into 4–5 μm thick slices using a microtome. After deparaffinization and rehydration, the sections were stained with hematoxylin and eosin (H&E) for histological evaluation. Finally, the stained sections were examined and imaged under a microscope. All procedures were performed according to established methods [21].

### 2.10. Immunohistochemistry

After dewaxing paraffin sections of jejunum tissues, antigen retrieval and blocking were conducted. The primary antibody (anti-mouse CD68 antibody) diluted 1:500 was added, incubated at room temperature for 60 min and then at 4 °C overnight. The next day, biotin-labeled secondary antibody was added and incubated for 30 min, followed by dropwise addition of DAB solution. After counterstaining, dehydration, clearing and mounting, the sections were observed and photographed under a microscope [22].

### 2.11. Immunostaining

After dewaxing the paraffin sections of intestinal tissue, antigen retrieval, permeabilization, and blocking were performed. The primary antibodies (anti-mouse CD68 and CD86) were added at a dilution of 1:500 and incubated overnight at 4 °C. The following day, fluorescently labeled secondary antibodies were added and incubated for 1 h. After counterstaining and mounting, the sections were observed and photographed under a fluorescence microscope. The protein expression levels in each group were analyzed using Image-Pro Plus 6.0 software [23].

### 2.12. Statistical Analysis

All experiments were carried out independently a minimum of three times. Image J software (version 1.53k) was utilized to quantify the grayscale values of proteins. Data were expressed as mean ± standard deviation (SD). One-way ANOVA or unpaired *t*-test was employed for significance analysis, and GraphPad Prism 8 was used to create statistical graphs. Significance levels (*p*) were marked as follows: *** for *p* < 0.001, ** for *p* < 0.01, * for *p* < 0.05, and n.s. for no significant difference [24].

## 3. Results

### 3.1. Taurine Inhibits M1 Macrophage Inflammation

**Figure 1:**
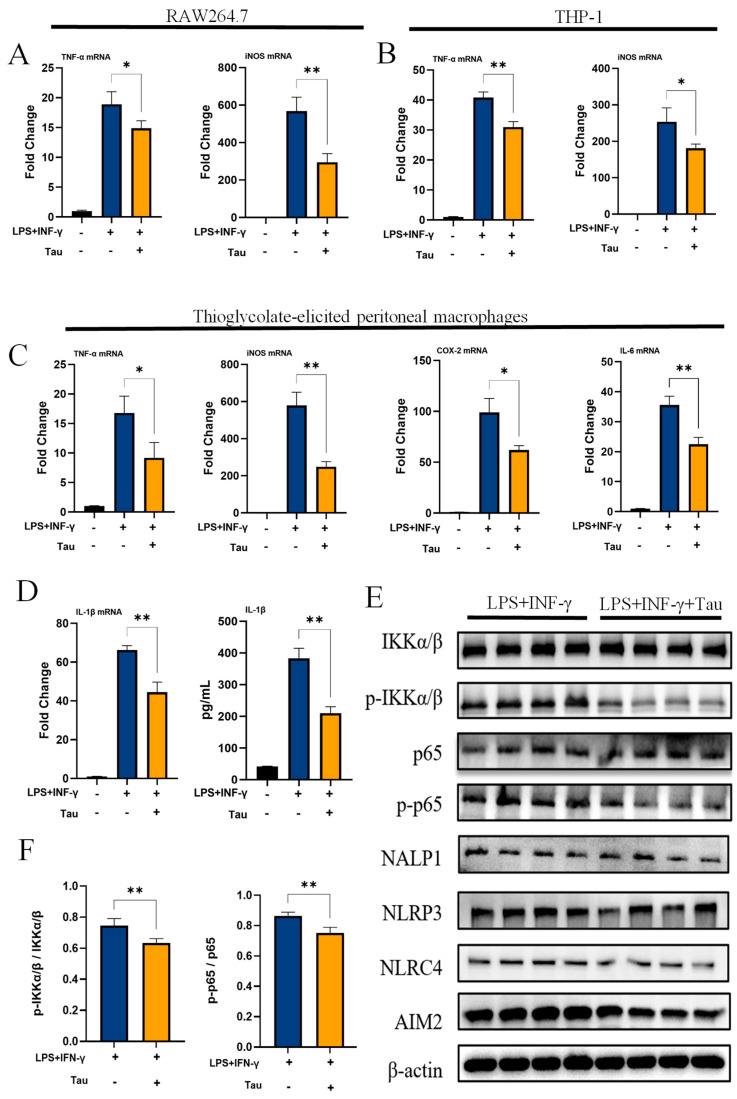
Taurine inhibits M1 macrophage inflammation. (**A**) mRNA expressions in RAW264.7 cells. (**B**) mRNA expressions in THP-1 cells. (**C**) mRNA expressions in TGPM cells. (**D**) The secretion and mRNA expression of IL-1β from TGPMs. (**E**) Western blot showing the protein abundance of IKKα/β, p-IKKα/β, p65, p-p65, NALP1, NLRP3, NLRC4 and AIM2 in TGPMs. (**F**) Statistical analysis of the activation of NF-κB in different groups (*n* = 4). Data were analyzed with one-way ANOVA. All results shown are representative of three independent experiments with n = 4 per group and shown as the means ± SD. (*, *p* < 0.05; **, *p* < 0.01).

Taurine significantly suppressed M1 polarization in RAW264.7 and THP-1 cells, reducing TNF-α (*p* = 0.046 and *p* = 0.0029) and iNOS (*p* = 0.0058 and *p* = 0.034) mRNA levels (Figure 1A,B). In primary TGPMs, taurine downregulated TNF-α (*p* = 0.026), iNOS (*p* = 0.0017), COX-2 (*p* = 0.011), and IL-6 (*p* = 0.0036) mRNA levels (Figure 1C). It also inhibited IL-1β at transcriptional (*p* = 0.0026) and secretory (*p* = 0.0013) levels (Figure 1D). Since M1 macrophage function involves the activation of intracellular signaling pathways, such as NF-κB and inflammasomes, we analyzed the activation status of these pathways. Taurine reduced p-IKK (*p* = 0.0058) and p-p65 (*p* = 0.0022) phosphorylation, but had no significant effect on NALP1, NLRP3, NLRC4 and AIM2 (Figure 1E,F). Furthermore, treatment of M0 macrophages with taurine alone did not directly induce M2 polarization (Figure S1). Taken together, these results demonstrated that taurine can inhibit the inflammatory response of M1.

### 3.2. Taurine Induces Metabolic Reprogramming in M1 Macrophages

**Figure 2:**
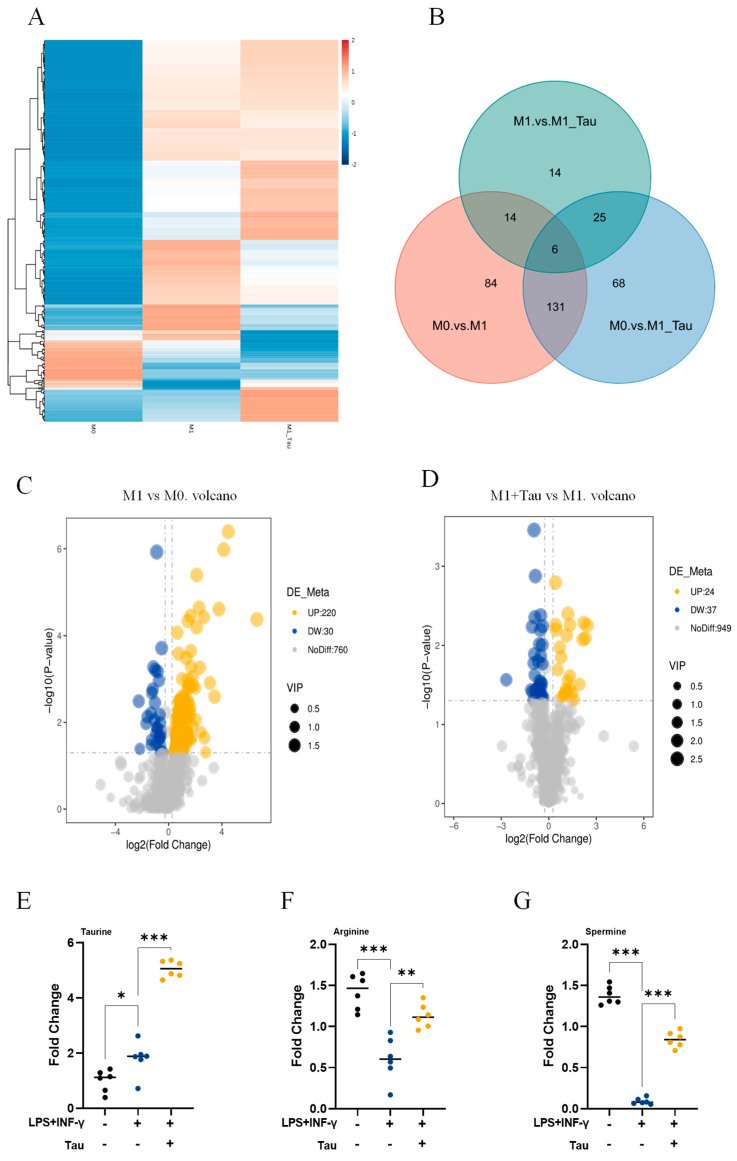
Taurine induces metabolic reprogramming in M1 macrophages. (**A**) Clustering heatmap of total differential metabolites. (**B**) Venn diagram of differential metabolites. (**C**) Volcano plot showing the differential variables between the M1 and M0 groups. (**D**) Volcano plot showing the differential variables between the M1+Tau and M1 groups. (**E**–**G**) Fold change of intracellular metabolites: taurine, arginine and spermine (*n* = 6). Data were analyzed by one-way ANOVA and are shown as the means ± SD. (*, *p* < 0.05; **, *p* < 0.01; ***, *p* < 0.001).

Metabolomic profiling revealed that M1 polarization triggered substantial metabolic reprogramming, with 220 metabolites significantly elevated and 20 significantly decreased compared to the M0 state. Taurine co-treatment markedly inhibited this process, as evidenced by the identification of 14 unique differential metabolites, indicating that taurine can partially inhibit the metabolic phenotype transition from M0 to M1 macrophages (Figure 2A–D). Consistent with this inhibition, taurine supplementation significantly enhanced intracellular levels of taurine (*p* < 0.001), arginine (*p* = 0.002), and its downstream metabolite spermine (*p* < 0.001) (Figure 2E–G). Interestingly, intracellular taurine levels were significantly elevated in M1 macrophages even without taurine supplementation (*p* = 0.022). Collectively, these findings demonstrate that by modulating key metabolites, taurine reprograms macrophage metabolism to attenuate inflammatory responses.

### 3.3. Taurine Inhibits IL-1β Production in M1 Macrophages by Suppressing the JAK1/2-STAT1 Signaling Pathway

**Figure 3:**
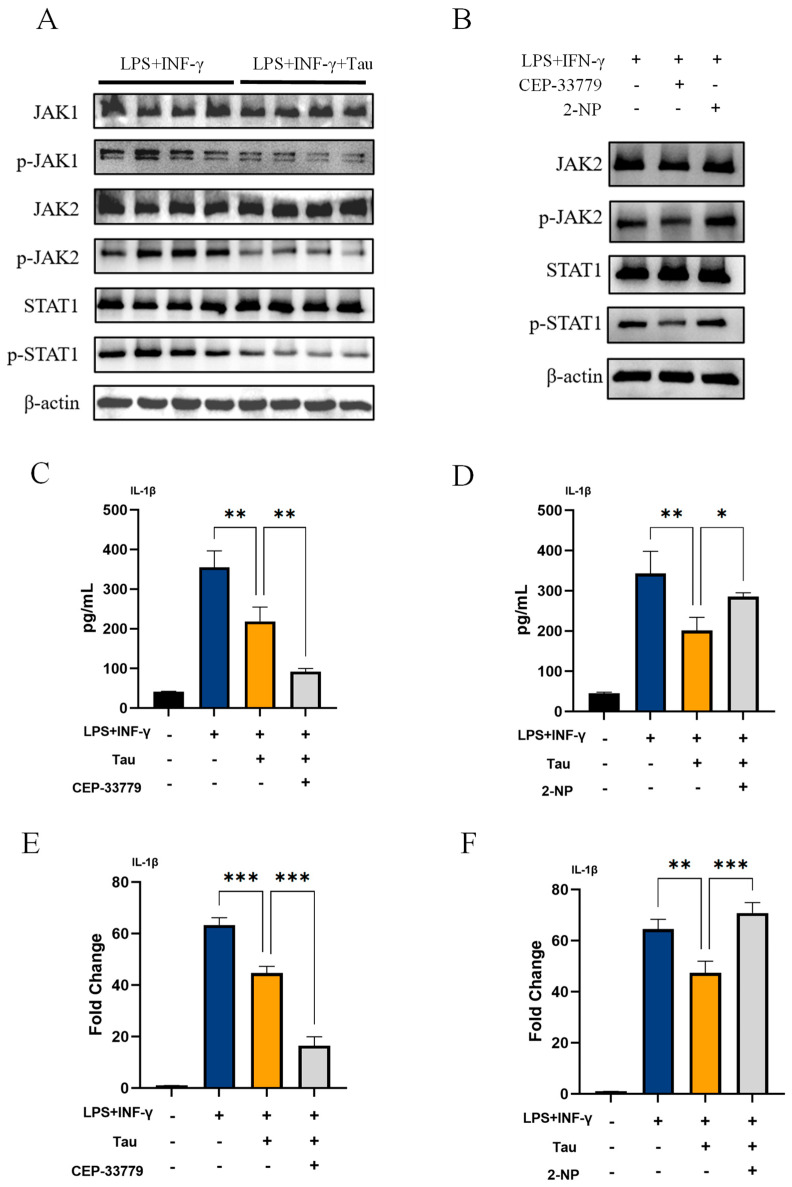
Taurine inhibits IL-1β production in M1 macrophages by suppressing the JAK1/2-STAT1 signaling pathway. (**A**) Western blot analysis of JAK1/2-STAT1 pathway proteins in TGPMs. (**B**) Western blot analysis of JAK2 and STAT1 phosphorylation under different treatments. (**C**,**D**) IL-1β secretion and (**E**,**F**) mRNA expression in TGPMs. For inhibitor and activator treatments, cells were pretreated for 1 h with 1 µM CEP-33779 (JAK2 inhibitor) or 45 µM 2-NP (STAT1 activator) prior to M1 polarization with LPS/IFN-γ in the presence or absence of taurine. Data were analyzed by one-way ANOVA. All results shown are representative of three independent experiments and are presented as mean ± SD. (*, *p* < 0.05; **, *p* < 0.01; ***, *p* < 0.001).

Given that taurine upregulates spermine, a known inhibitor of JAK1/2–STAT1 signaling [25], we investigated its role in taurine’s anti-inflammatory effect. We found that taurine significantly suppressed JAK1/2–STAT1 activation in M1 macrophages (Figure 3A). Consistent with this, treatment with the JAK2 inhibitor CEP-33779 [26,27] decreased both IL-1β secretion (*p* = 0.0024) and mRNA expression (*p* < 0.001) (Figure 3B,C), whereas treatment with the STAT1 activator 2-NP [28] reversed the taurine-induced suppression of IL-1β secretion (*p* = 0.0496) and mRNA expression (*p* < 0.001) (Figure 3B,D). Taken together, these results demonstrate that taurine inhibits IL-1β production in M1 macrophages by suppressing the JAK1/2–STAT1 pathway.

### 3.4. Taurine Alleviates Systemic Inflammation In Vivo

**Figure 4:**
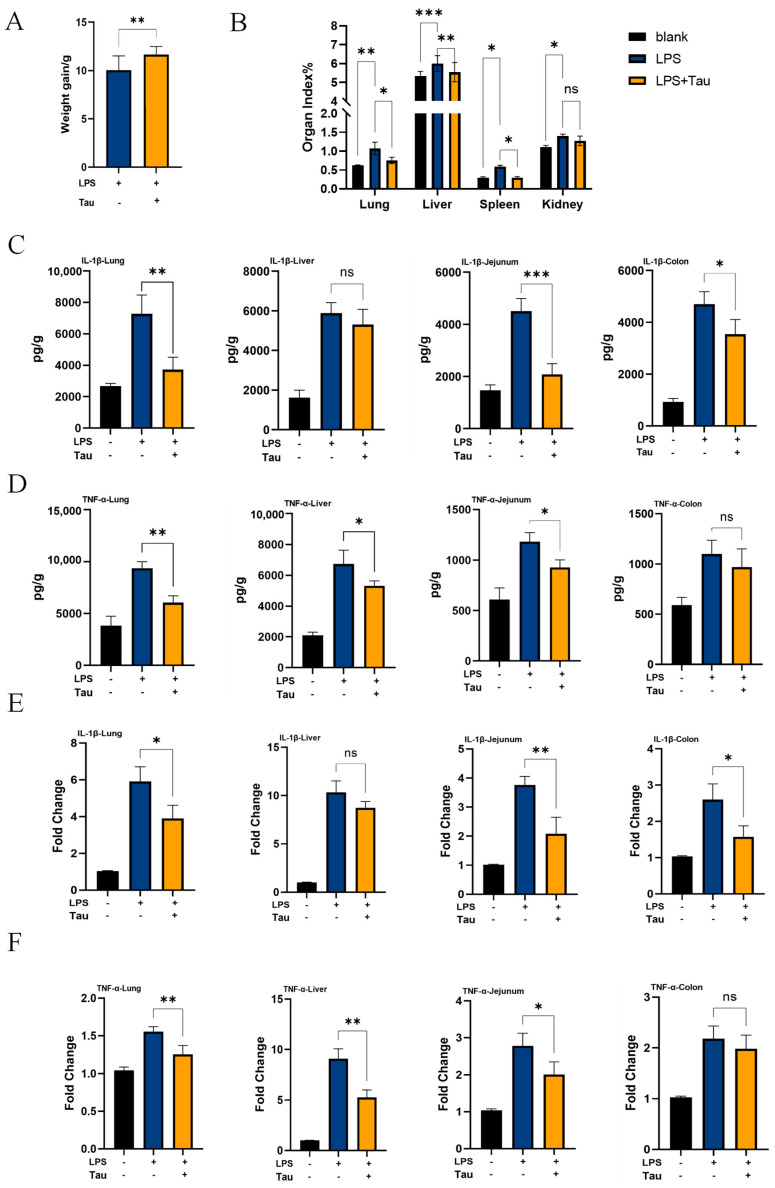
Taurine alleviates systemic inflammation in vivo. (**A**) Body weight gain of mice in control and taurine-supplemented groups (*n* = 8). (**B**) Organ indices of lung, liver, spleen, and kidney (*n* = 8). (**C**,**D**) Levels of IL-1β and TNF-α in lung, liver, jejunum, and colon (*n* = 8). (**E**,**F**) mRNA expression of IL-1β and TNF-α in the indicated organs. Data were analyzed by unpaired *t*-test (**A**) or one-way ANOVA (**B**–**F**) and are shown as the mean ± SD. (n.s., *p* > 0.05; *, *p* < 0.05; **, *p* < 0.01; ***, *p* < 0.001).

To investigate whether taurine supplementation alleviates inflammation in vivo, we administered taurine in the drinking water of 3-week-old mice for 4 weeks. Taurine-supplemented mice showed significantly increased weight gain compared to controls (*p* = 0.0033) (Figure 4A). Following intraperitoneal injection of LPS (10 mg/kg) to induce sepsis, mice developed characteristic clinical signs including piloerection, lethargy, and huddling behavior. In contrast, mice in the taurine-supplemented group exhibited a significant alleviation of these symptoms. The organ indices of the lung (*p* = 0.03), liver (*p* = 0.0056), and spleen (*p* = 0.041) were lower in the taurine-supplemented group than in the LPS group, whereas the kidney index was unaffected (*p* = 0.62) (Figure 4B). Consistent with these findings, taurine supplementation reduced IL-1β protein content in the lung (*p* = 0.0048), jejunum (*p* < 0.001), and colon (*p* = 0.041) (Figure 4C), and decreased TNF-α protein content in the lung (*p* = 0.0036), liver (*p* = 0.048), and jejunum (*p* = 0.035) (Figure 4D). Furthermore, taurine significantly attenuated the LPS-induced upregulation of IL-1β and TNF-α mRNA levels in multiple organs (Figure 4E,F). Collectively, these results demonstrate that taurine supplementation alleviates LPS-induced systemic inflammation in vivo.

### 3.5. Taurine Alleviates Histopathological Damage and Intestinal Macrophage Polarization

**Figure 5:**
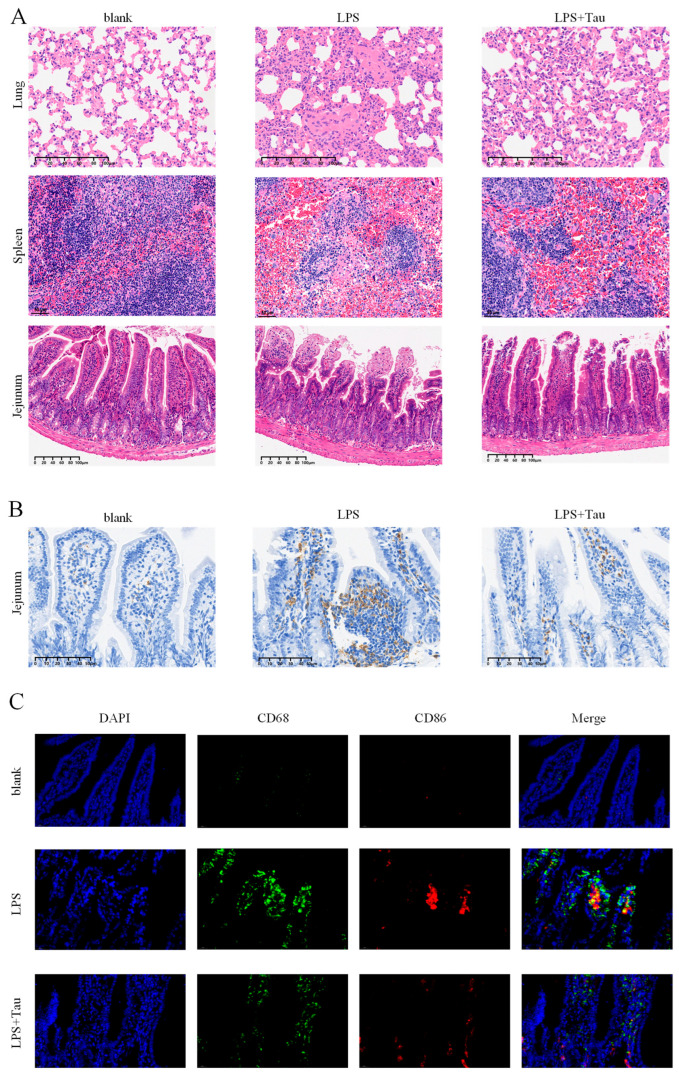
Taurine alleviates histopathological damage and intestinal macrophage polarization. (**A**) Hematoxylin and eosin staining of lung, spleen and jejunum in mice. (**B**) Immunohistochemical staining for CD68 in the jejunum. (**C**) Immunofluorescent staining for CD68 and CD86 in the jejunum. Green indicates CD68, and red indicates CD86.

Histopathological analysis demonstrated taurine’s protective effects against LPS-induced tissue injury across multiple organs. In the lung, taurine significantly attenuated characteristic injuries including neutrophilic infiltration, interstitial edema, alveolar wall thickening, and hyaline membrane formation, resulting in a markedly lower pathological score. In the spleen, taurine ameliorated white pulp hyperplasia, sinus expansion, substantial macrophage aggregation, and granuloma-like structure formation observed in LPS-treated mice. In the jejunum, taurine alleviated villus blunting, crypt deepening, focal epithelial loss, and edema in the mucosa and submucosa, leading to an improved villus height-to-crypt depth ratio (Figure 5A). Furthermore, taurine suppressed macrophage infiltration and M1 polarization in the jejunum, as shown by reduced CD68^+^ cells via immunohistochemistry (Figure 5B) and decreased co-expression of CD68 and the M1 marker CD86 via immunofluorescence (Figure 5C).

## 4. Discussion

Macrophage over-polarization to the M1 phenotype is a key driver of inflammatory diseases. Modulating this process via taurine, the most abundant free amino acid in macrophages, offers a fresh therapeutic perspective. While taurine’s broad anti-inflammatory effects—such as osmotic regulation, inhibition of macrophage recruitment, and suppression of pro-inflammatory cytokine secretion—have been widely reported [29,30,31,32,33,34,35,36], our work identifies a previously unexplored role: taurine acts as a “metabolic signal converter” that promotes spermine accumulation to precisely control upstream inflammatory signaling, thereby shifting the understanding of its function from descriptive observations to mechanistic insights.

Our investigation into the mechanism began with an expected observation: taurine suppressed the expression of multiple inflammatory cytokine genes and the activation of the classic NF-κB pathway in M1 macrophages. However, metabolomic profiling revealed a more instructive mechanism involving significant reprogramming of amino acid metabolism that led to substantial spermine accumulation. Given spermine’s known function as an endogenous JAK1 inhibitor, we subsequently demonstrated that taurine potently inhibits the JAK1/2-STAT1 pathway—the canonical cascade driving M1 polarization through nuclear translocation of phosphorylated STAT1 dimers that activate M1-associated genes while suppressing M2-polarizing factors [37,38]. This defines a new regulatory pathway where taurine-induced spermine accumulation specifically curbs the JAK1/2-STAT1 axis to reduce M1 polarization and IL-1β production. The role of spermine in IL-1β inhibition is further supported by evidence from multiple disease models. Specifically, spermine has been shown to reduce IL-1β in acute liver injury, suppress NLRP3 inflammasome activation in diabetic atherosclerosis, and downregulate Guanylate Binding Protein 5 (GBP5)-mediated NLRP3 activation during viral infection [39,40,41]. Our discovery attests to a core feature of immune regulation: the induction of negative regulators like spermine to actively constrain excessive inflammation. This same feature is similarly embodied in the amidase-mediated feedback loop recently identified in invertebrates [42]. Another noteworthy finding is that M1 polarization itself triggers an adaptive response, increasing intracellular taurine levels. However, this self-regulated taurine boost is insufficient to effectively curb M1 polarization and IL-1β secretion, indicating it is an inadequate compensatory response. This further highlights the essential value of supplementing exogenous taurine to achieve higher therapeutic concentrations in treatment strategies. To definitively establish spermine’s essential role, our subsequent investigations will employ ornithine decarboxylase inhibitors to block its synthesis and evaluate the impact on taurine’s effects.

The mechanism we identified aligns with a recent study showing that taurine restricts S-adenosylmethionine (SAM) availability, inhibits Protein Phosphatase 2A Catalytic subunit α isoform (PP2Ac) methylation, and thereby blocks the shift between mitophagy and glycolysis [43]. We propose that these two pathways function synergistically: spermine accumulation delivers the key instruction to inhibit JAK/STAT signaling, while SAM restriction-induced metabolic reprogramming (such as impaired mitophagy and glycolysis inhibition) creates a suitable cellular environment for efficient execution of this instruction. For instance, the high mitochondrial density maintained by PP2Ac demethylation may enhance STAT1’s sensitivity to spermine-mediated inhibition by influencing acetyl-CoA levels to regulate STAT1 acetylation or altering the cellular redox state to modulate its phosphorylation activity [44,45,46]. Together, these pathways outline a “signaling-metabolism” cooperative network through which taurine regulates macrophage polarization. Translating this theoretical discovery into clinical applications, such as developing small-molecule drugs to precisely target specific nodes in this network, represents both a core value and a challenge for future research.

We recognize that the extensive metabolomic changes induced by taurine suggest the existence of a complex regulatory network. Beyond the spermine pathway, other immunomodulatory metabolites such as gamma-aminobutyric acid (GABA,) glutathione, and betaine may also participate and collectively contribute to the overall anti-inflammatory effect [47,48,49], representing a direction worthy of further exploration. Additionally, the significant protective effects observed in the gut point to additional systemic mechanisms. Studies have shown that oral taurine can reshape the gut microbiota. The underlying mechanism involves taurine intake increasing the levels of taurine-conjugated bile acids in the intestine, thereby providing a growth advantage for bacteria capable of metabolizing taurine or tolerating bile acids. For instance, several beneficial bacteria such as Bifidobacterium and Lactobacillus possess bile salt hydrolase (BSH) [50,51,52], which deconjugates bile acids, enabling these bacteria to utilize the released taurine as an energy source or for other physiological processes, thereby enhancing their colonization and proliferation. This is evidenced in a hyperuricemic nephropathy model by an increase in beneficial bacteria like *Lactobacillus*, and in LPS-challenged piglets by the alleviation of intestinal damage through the modulation of microbial populations and host-microbe co-metabolites [53,54].

Therefore, the notable anti-inflammatory effects observed in our mouse sepsis model may partly originate from taurine’s regulation of gut microbiota and their metabolites via the above pathways, working synergistically with the direct macrophage reprogramming mechanism identified in vitro. This understanding also directs future research: using germ-free or antibiotic-treated models to distinguish the contributions of direct cellular effects from microbe-mediated indirect effects, and to identify which microbiota-derived metabolites synergistically enhance taurine’s systemic anti-inflammatory action in vivo.

## 5. Conclusions

This study elucidates a novel anti-inflammatory mechanism of taurine, in which spermine plays a central role as a key mediating factor. Specifically, taurine-driven metabolic reprogramming leads to the specific accumulation of spermine (Figure 2E–G), which in turn inhibits the JAK1/2-STAT1 pathway (Figure 3A,B) and ultimately downregulates IL-1β production (Figure 3C–F). In vivo, taurine not only alleviated multi-organ inflammation and tissue damage in a septic mouse model (Figure 4B–F and Figure 5A) but also suppressed intestinal macrophage infiltration and M1 polarization (Figure 5B,C). By integrating theoretical insights with practical applications and employing both in vivo and in vitro approaches, this study deepens our understanding of taurine’s role in macrophage immunometabolism and thereby broadens its potential for clinical application.

## Figures and Tables

**Table 1:** Representative diseases associated with abnormal macrophage polarization.

Polarization State	Representative Diseases	Pathological Consequences
M1 Dominance	Sepsis, Atherosclerosis	Sustained inflammation, tissue damage
M2 Dominance	Cancer, Fibrotic diseases	Immunosuppression, tissue scarring

## Data Availability

Data will be made available on request.

## Supplementary Materials

The following supporting information can be downloaded at: https://www.mdpi.com/article/10.3390/biology14121751/s1, Figure S1: M0 macrophages were treated with 20 ng/mL IL-4 for 48 h or with 10 mM taurine only. Table S1. List of Abbreviations.
