# Supplementary material for: Taurine Attenuates M1 Macrophage Polarization and IL-1β Production by Suppressing the JAK1/2-STAT1 Pathway via Metabolic Reprogramming
Source: Biology (Basel). 2025 Dec 6;14(12):1751. doi: 10.3390/biology14121751 (PMC12730316; doi:10.3390/biology14121751)
Supplement: Supplementary file 1 [file biology-14-01751-s001.zip › biology-4009350 - Original Western Blots.pdf]

IKK for Fig 1E

70kDa

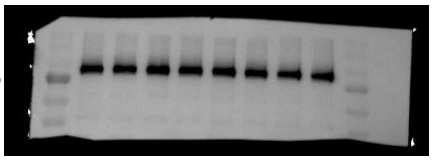

87kDa

p-IKK for Fig 1E

70kDa

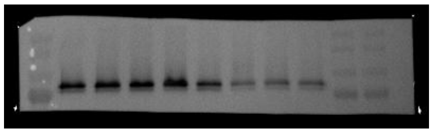

87kDa

p65 for Fig 1E

55kDa

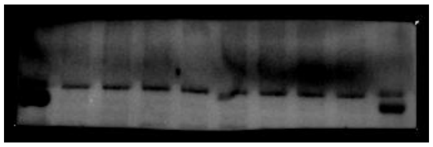

65kDa

p-p65 for Fig 1E

55kDa

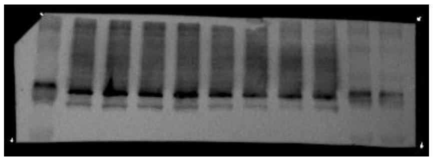

65kDa

NALP1 for Fig 1E

130kDa

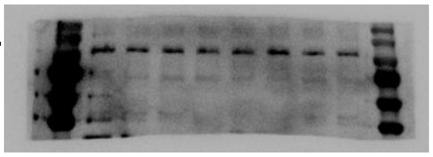

118kDa

NLRP3 for Fig 1E

130kDa

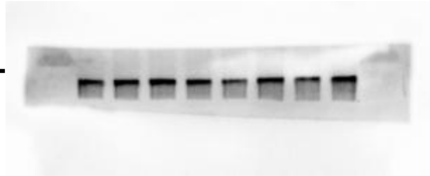

118kDa

NLRC4 for Fig 1E

130kDa

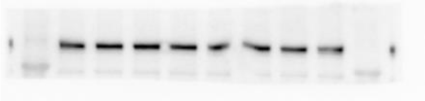

116kDa

AIM2 for Fig 1E

25kDa

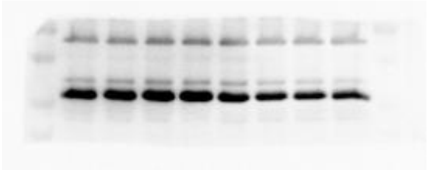

32kDa

β-actin for Fig 1E

40kDa

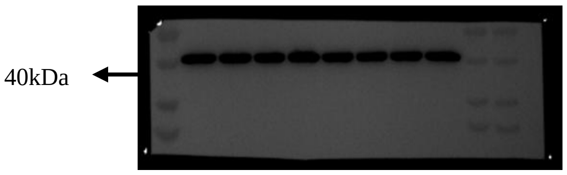

42kDa

JAK1 for Fig 3A

130kDa

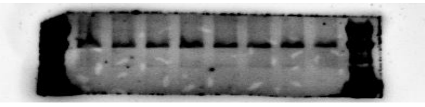

130kDa

p-JAK1 for Fig 3A

130kDa

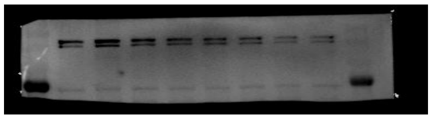

130kDa

JAK2 for Fig 3A

130kDa

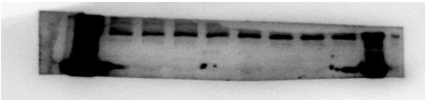

125kDa

P-JAK2 for Fig 3A

130kDa

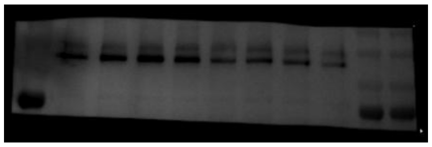

125kDa

STAT1 for Fig 3A

70kDa

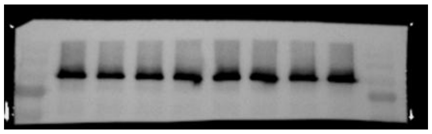

84kDa

P-STAT1 for Fig 3A

70kDa

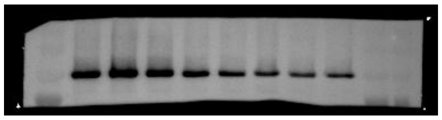

84kDa

β-actin for Fig 3A

40kDa

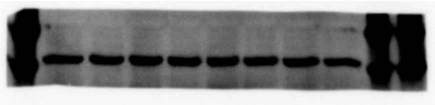

42kDa

JAK2 for Fig 3B

130kDa

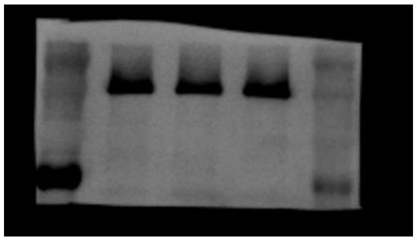

125kD
a

p-JAK2 for Fig 3B

130kDa

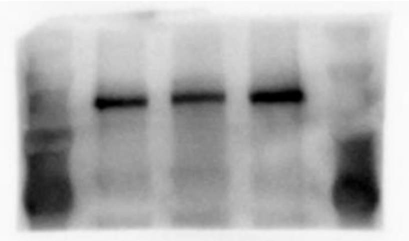

125kD
a

STAT1 for Fig 3B

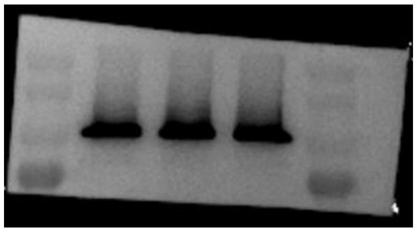

84kDa

70kDa

p-STAT1 for Fig 3B

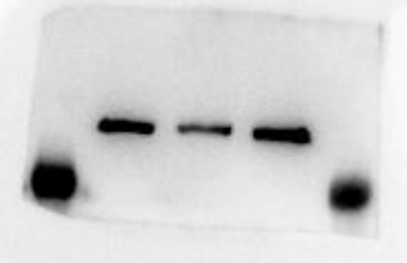

84kDa

70kDa

β-actin for Fig 3B

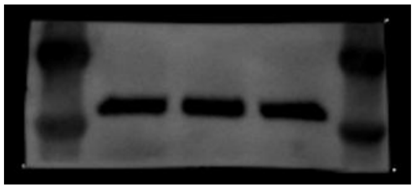

42kDa

40kDa
