# Supplementary material for: Taurine Attenuates M1 Macrophage Polarization and IL-1β Production by Suppressing the JAK1/2-STAT1 Pathway via Metabolic Reprogramming
Source: Biology (Basel). 2025 Dec 6;14(12):1751. doi: 10.3390/biology14121751 (PMC12730316; doi:10.3390/biology14121751)
Supplement: Supplementary file 1 [file biology-14-01751-s001.zip › biology-4009350 Supplementary Materials.pdf]

Figure S1

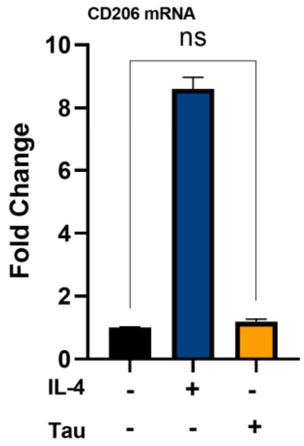

Figure S1: M0 macrophages were treated with 20 ng/mL IL-4 for 48 hours or with 10 mM taurine only.

Table S1

List of Abbreviations

Abbreviation	English Full Name
IL-1β	Interleukin-1β
LPS	Lipopolysaccharide
IFN-γ	Interferon-γ
JAK1/2-STAT1	Janus Kinase1/2-Signal Transducer and Activator of Transcription 1
IL-4	Interleukin-4
Ym1	Chitinase-like protein 3
IL-10	Interleukin-10
TNBS	2,4,6-Trinitrobenzene sulfonic acid
MPO	Myeloperoxidase
HOCl	Hypochlorous Acid
TauCl	Taurine chloramine
NF-κB	Nuclear Factor-κB

mTOR	Mammalian Target of Rapamycin
iNOS	Inducible Nitric Oxide Synthase
mTORC1	Mammalian Target of Rapamycin Complex 1
Acetyl-CoA	Acetyl coenzyme A
TGPMs	Thioglycolate-Elicited Peritoneal Macrophages
PBS	Phosphate-Buffered Saline
PMA	Phorbol 12-Myristate 13-Acetate
TNF-α	Tumor Necrosis Factor-α
COX-2	Cyclooxygenase-2
IL-6	Interleukin-6
CEP-33779	JAK2 selective inhibitor (CEP-33779)
2-NP	2-Naphthyl phosphate
GBP5	Guanylate-Binding Protein 5
SAM	S-adenosylmethionine
PP2Ac	Protein Phosphatase 2A Catalytic subunit
GABA	γ-Aminobutyric Acid
BSH	Bile Salt Hydrolase
